# The Osteoinductivity of Calcium Phosphate-Based Biomaterials: A Tight Interaction With Bone Healing

**DOI:** 10.3389/fbioe.2022.911180

**Published:** 2022-05-16

**Authors:** Yuchen Zhang, Tianyu Shu, Silin Wang, Zhongbo Liu, Yilong Cheng, Ang Li, Dandan Pei

**Affiliations:** ^1^ Key Laboratory of Shaanxi Province for Craniofacial Precision Medicine Research, College of Stomatology, Xi’an Jiaotong University, Xi’an, China; ^2^ School of Chemistry, Xi’an Jiaotong University, Xi’an, China

**Keywords:** calcium phosphate, biomaterials, osteoinductivity, biodegradation, bone healing

## Abstract

Calcium phosphate (CaP)-based bioceramics are the most widely used synthetic biomaterials for reconstructing damaged bone. Accompanied by bone healing process, implanted materials are gradually degraded while bone ultimately returns to its original geometry and function. In this progress report, we reviewed the complex and tight relationship between the bone healing response and CaP-based biomaterials, with the emphasis on the *in vivo* degradation mechanisms of such material and their osteoinductive properties mediated by immune responses, osteoclastogenesis and osteoblasts. A deep understanding of the interaction between biological healing process and biomaterials will optimize the design of CaP-based biomaterials, and further translate into effective strategies for biomaterials customization.

## 1 Introduction

The clinical intervention of bone healing is necessary for bone defects beyond the critical size, such as large segmental bone defects caused by severe trauma, tumor resection, cancer, congenital diseases, or small-scale alveolar bone defects (Davies et al., 2017; Majidinia et al., 2018). Given inevitable drawbacks of autografts (including donor site pain, increased operative time, risk of wound infection, and insufficient availability), many synthetic biomaterials have been specifically designed as an alternative for bone repair and regeneration (Pina et al., 2015; Xu et al., 2017; Stastny et al., 2019). Since the 1970s, calcium phosphate (CaP)-based bioceramics have received the most attention, and several of them [e.g., β-tricalcium phosphate (β-TCP) and hydroxyapatite (HA or HAP)] have already been clinically applied for orthopedic and maxillofacial surgery (Schaefer et al., 2011; Danoux et al., 2015).

For bone-filling purposes, CaPs have been utilized as bioactive components of solid ceramic, coatings, self-setting CaP cements (CPC), as well as advanced polymers, intending to function as a scaffold for bone formation (Parent et al., 2017). The family of CaP biomaterials comprises varying phase compositions, including HA [Ca_10_(PO_4_)_6_(OH)_2_], tricalcium phosphate [TCP, Ca_3_(PO_4_)_2_], octacalcium phosphate [OCP, Ca_8_(HPO_4_)_2_(PO_4_)_4_·5H_2_O], dicalcium phosphate dihydrate [DCPD, CaHPO_4_.2H_2_O], and amorphous calcium phosphate [ACP, CaxHy(PO_4_)z·nH_2_O, *n* = 3–4.5; 15–20% H_2_O], can be used in a variety of applications due to differences in solubility, stability, and mechanical strength (Jeong et al., 2019; Dorozhkin, 2021). CaP-based biomaterials provide a strong biomaterial/bone interface and demonstrate promising biological properties such as biodegradability, osteoconductivity, and in some cases even osteoinductivity (i.e., the ability of the material to induce *de novo* bone formation without the presence of osteogenic factors) (LeGeros, 2008; Chai et al., 2012; Stastny et al., 2019). In addition, CaPs can be manufactured in large quantities at relatively low cost, meeting the requirements of socially responsible tissue engineering and regenerative medicine (Habraken et al., 2016).

Much effort has been spent on optimizing biomechanical and physico-chemical properties to “engineer” materials for better “osteoinductivity” (Bigi and Boanini, 2017; Ruffini et al., 2021). In this context, the stiffness, porosity and pore size, surface microstructure, phase composition, and crystallinity have all been shown to affect the bone regeneration capacity of CaP-based materials. These bone-matching “static” physico-chemical parameters and the underlying osteogenesis mechanisms have been summarized in recent reviews for material design optimization (Habraken et al., 2016; Galvan-Chacon and Habibovic, 2017; Przekora, 2019; Dee et al., 2020). Nevertheless, the laboratory-prepared bone graft materials are often not successful in clinical application (Chen Z. et al., 2016). The regeneration capacity of biomaterials is usually evaluated by *in vitro* osteoblastic lineage (Diez-Escudero et al., 2017b), and *in vivo* bone histomorphology measurements (Yuan et al., 2000; Chiba et al., 2016). However, the biological process of bone healing to implanted materials has always been neglected. The mechanisms underlying bone healing are not simply led by osteoblasts, but a synergy of multiple systems involving a series of biological events including early inflammation response and long-term reconstruction process (Newman et al., 2021; Zhu et al., 2021). Therefore, the optimization and design of CaP-based biomaterials should keep pace with the rapid progress in bone healing biology, which eventually determines the *in vivo* fate of CaP-based biomaterials and bone healing effects.

In this review, we first summarized the biological process of bone healing and then focused on major developments in the bone healing response to CaP-based biomaterials. Understanding the interaction between biomaterials and host response during the bone healing process is important for improving the design of CaP-based biomaterials. Simultaneously, we also provide an outlook toward customizable CaP-polymer composite biomaterial strategies.

## 2 Overview of Bone Healing Process

There are three overlapping stages of the bone healing process: inflammation, bone formation and bone remodeling (Maruyama et al., 2020; Newman et al., 2021; Zhu et al., 2021).

Inflammation begins immediately after the bone is broken and lasts for several days. Driven by cytokines, macrophages and other immune cells (granulocytes, lymphocytes, monocytes, etc.) infiltrate bone defects, which trigger inflammatory reactions, clean up bone-tissue debris, as well as form vascular tissue and granulation tissue (Maruyama et al., 2020). Macrophages demonstrate broad roles here in regulating bone tissue homeostasis. Under the stimulation of the locally ischemic and hypoxic environment, and cytokines [e.g., macrophage colony-stimulating factor (M-CSF) and receptor activator of NF-κB ligand (RANKL)], macrophages polarize towards “M1” type. They secrete a series of pro-inflammatory cytokines [e.g., tumor necrosis factor-α (TNF-α), interleukin-1 (IL-1), IL-6], which in turn recruit mesenchymal stem cells (MSCs), osteoprogenitor cells, and fibroblasts for tissue repair (Miron et al., 2016; Zhu et al., 2021). Macrophages transform towards M2 wound-healing type at the end of the inflammatory stage and secrete anti-inflammatory cytokines [e.g., IL-4, IL-10, and transforming growth factors beta (TGF-β)] in favor of establishing osteogenic environments (Oliveira et al., 2021).

During the bone formation process, there are two repair mechanisms depending on the location of the bone defect. The cancellous and inter-cortical bone regions are repaired by endochondral ossification, while the subperiosteal and adjacent soft tissue regions are repaired by intramembranous ossification (Zhu et al., 2021). In cancellous and inter-cortical bone regions, the recruited MSCs aggregate, proliferate and differentiate into chondrocytes. They secrete an avascular cartilage matrix, and the granulation tissue is gradually replaced by the fibrocartilage- and hyaline cartilage-rich soft callus (Nossin et al., 2021). Along with new blood vessel formation, endothelial cells, osteoblasts, and chondrocytes secrete matrix metalloproteinases to degrade the cartilage matrix. Mature hypertrophic chondrocytes undergo apoptosis or transition into osteoblast-like cells, which together with osteoblasts, contribute to the secretion of type I collagen and the extracellular matrix (ECM) mineralization. Finally, the soft callus is transformed into the disordered woven bone (Tsang et al., 2015; Fu et al., 2021). Once the periosteum and adjacent soft tissue regions are injured, the MSCs recruited from the periosteum, bone marrow, adjacent soft tissues, and peripheral circulation and osteoprogenitor cells within the periosteum initiate the intramembranous bone formation process. MSCs and osteoprogenitor cells differentiate into osteoblasts. These cells secrete and mineralize ECM, forming hard callus directly under the periosteum (Wang et al., 2020).

Bone remodeling is the final stage of bone healing and typically lasts for several months (ElHawary et al., 2021). As a dynamic process, the irregular woven bone is reconstructed by bone resorption and osteogenesis process under mechanical stimulation and further developed as mature lamellar bone (Frost, 2004; Loi et al., 2016; ElHawary et al., 2021). The crosstalk between osteoblasts and osteoclasts plays an important role in this phase. Bone ultimately returns to its original shape and restores the geometry and function of the damaged part (Zhu et al., 2021).

## 3 Calcium Phosphate-Based Biomaterials Participate in Bone Healing Process

**FIGURE 1:**
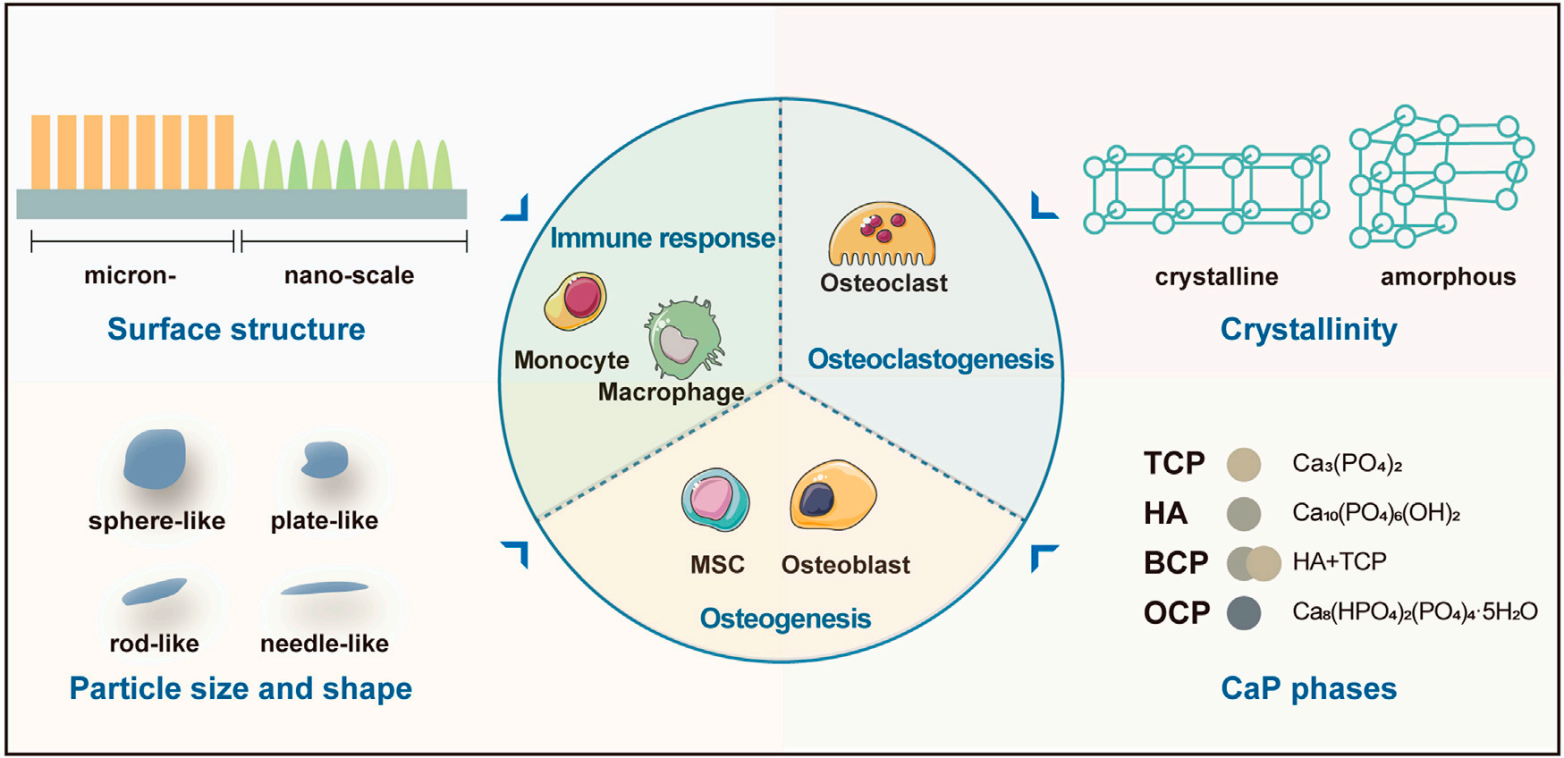
The physico-chemical properties of CaP-based biomaterials affect the activity and function of immune cells, osteoclasts, and osteoblasts. Abbreviation: BCP, biphasic calcium phosphate; TCP, tricalcium phosphate; HA, hydroxyapatite; MSC, mesenchymal stem cell; OCP, octacalcium phosphate.

With similar composition to bone minerals, CaP-based biomaterials induce biological responses like those occur during bone healing (Diez-Escudero et al., 2017a). On the one hand, multiple cells involved in bone healing mediate the degradation and resorption of implanted materials. This is critical for CaP-based biomaterials to provide the space into which the bone and vascular tissues can grow (Rustom et al., 2019). The concomitant release of inorganic ions from the biodegradation of CaPs is also necessary for modifying the behavior of cells/tissues and enhances the osteoinductivity of the biomaterials (Tang et al., 2018). On the other hand, the osteoinductivity of CaP-based biomaterials is complicated *in vivo* as inflammation and osteoclastogenesis accompany the entire process of bone healing. Recent studies have pointed out that the physico-chemical properties of CaP-based biomaterials could affect the activity and function of immune cells and osteoclasts, and indirectly or directly affect osteogenesis, respectively (Figure 1). In this section, we will detail these tight and complex interactions between CaP-based biomaterials and bone healing responses.

### 3.1 Mechanisms of *in vivo* Biodegradation of Calcium Phosphate-Based Biomaterials

After implantation, the host body will first undergo a universal immune response to the materials. Proteins (e.g., fibrinogen, vitronectin, complement, and fibronectin) from blood and interstitial fluid will immediately adsorb on the material surface and then trigger an acute inflammation. Chemoattractants and cytokines such as platelet factor 4 (PF-4), platelet-derived growth factor (PDGF), and TGF-β lead to monocyte colonization and differentiate into macrophages and osteoclasts (Chen Z. et al., 2016; Galvan-Chacon and Habibovic, 2017). Meanwhile, CaP-based biomaterials will undergo degradation and resorption processes *in vivo* (Sheikh et al., 2015). Degradation is a physical process utilizing chemical dissolution or mechanical degradation. The disintegration and fragmentation of implants are often accompanied by the release of material particles (Sheikh et al., 2015; Galvan-Chacon and Habibovic, 2017). Biomaterial degradation paths the way for cell-mediated resorption processes that involve macrophages, osteoclasts, and multinucleated giant cells (Sheikh et al., 2015; Miron and Bosshardt, 2016; Xiao et al., 2016).

CaP particles can be directly engulfed *via* phagocytosis and intracellular digestion by macrophages and osteoclasts (size <10 μm), as well as giant cells (size between 10 and 100 μm) (Heymann et al., 2001; Wenisch et al., 2003; Nicolin et al., 2016). The particles, if larger than 100 μm, will undergo extracellular degradation within the acidic microenvironment created by osteoclasts (Galvan-Chacon and Habibovic, 2017). Solubility of the biomaterial (Yamada et al., 1997; Detsch et al., 2010; Wu and Uskokovic, 2016) and topographic characteristics such as specific surface area and the porosity content (Zimmer et al., 2013; Davison et al., 2014; Diez-Escudero et al., 2017b) are relevant physicochemical factors that influence the osteoclasts resorption activity.

In addition, cytokines released by monocytes/macrophages are responsible for the recruitment of osteoprogenitor cells and MSCs (Przekora, 2019). Cytokines such as M-CSF, RANKL and osteoprotegerin are responsible for stimulating osteoclastogenesis (Chen Z. et al., 2016). Contrary to previous beliefs that all inflammatory responses should be suppressed after biomaterial implantation, healing is promoted by suppression of extended chronic inflammatory responses (Miron and Bosshardt, 2016; Batool et al., 2021).

### 3.2 The Interaction Between Bone Healing Response and Calcium Phosphate-Based Biomaterials

#### 3.2.1 Calcium Phosphate-Based Materials Regulate Osteoimmune

The concept of “osteoimmunomodulation” was proposed by Chen Z. et al. (2016) to emphasize the important roles of bone immune response in determining the *in vivo* fate of bone substitute materials. Macrophages are major effector cells in determining the duration and intensity of material-induced immune responses and are indispensable for osteogenesis (Chen et al., 2014a; Xiao et al., 2021). Chen and his team (Chen et al., 2014b) demonstrated that macrophages switch to the M2 phenotype through the calcium-sensing receptor (CaSR) pathway in response to β-TCP powder extracts. At the same time, the secreted bone morphogenetic protein 2 (BMP-2), as well as anti-inflammatory genes (IL-10 and IL-1rα) were up-regulated, and inflammatory genes (IL-1β and IL-6) were significantly down-regulated in macrophages. When bone marrow mesenchymal stem cells (BMSCs) were cultured in macrophage/β-TCP extracts conditioned medium, the osteogenic differentiation of BMSCs was significantly enhanced. More recent studies also reported similar findings that under the stimulation of biphasic calcium phosphate (BCP) ceramics, the expression of anti-inflammatory cytokine (IL-10) and growth factors, including vascular endothelial growth factor (VEGF), PDGF, epidermal growth factor (EGF), BMP-2 and TGF-β1 was increased, which might synergistically create a pro-osteogenic micro-environment, resulting in MSCs recruitment and differentiation into osteoblasts (Chen X. et al., 2016; Wang et al., 2017). Compared with macrophage/BCP conditioned media, the degradation particles of BCP might play a more substantial role in this process (Wang et al., 2017). However, the increased expression of inflammatory factors [IL-6, monocyte chemoattractant protein-1 (MCP-1), and TNF-α] in macrophages was found by Wang et al. (2017), and the increment of these pro-inflammatory cytokines did not seem to inhibit the osteogenic differentiation of MSCs. The reason may be that the effects of inflammatory cytokines are dose-dependent, e.g., only IL-6 of high concentration (above 10 ng ml^−1^) was proven to exhibit an inhibitory effect on BCP-induced osteogenesis (Chen X. et al., 2016). Further, more *in vivo* evaluations are needed for validating the immune-stimulated osteogenesis after CaP-based biomaterials implantation.

#### 3.2.2 Calcium Phosphate-Based Materials Regulate Osteoclast-Mediated Osteogenesis

Bone resorption mediated by osteoclasts is tightly coupled with osteogenesis of osteoblasts (Chen et al., 2018; Kim et al., 2020). Substantial efforts have been made in modifying the properties of CaP-based materials to regulate the activities of osteoclasts. And the phases, surface structure, and crystallinity of CaP materials have been shown to control osteoclast activities (Takami et al., 2009; Davison et al., 2014; Uskokovic et al., 2019; Wang et al., 2021). Wang et al. (2021) prepared a series of CPC scaffolds with different calcium and phosphate ratios (Ca/P) and found that slight release of phosphate ions from CPC with higher Ca/P ratio (1.67) improved osteoclastogenesis. The released phosphate ions promoted osteoclast differentiation *via* increased affinity between RANKL and RANK, as well as robust NF-κB signaling transduction. Rat calvarial defect model further supported that CPC with high Ca/P ratio could ameliorate osteoclast-mediated bone healing. In addition to ions effects, the topographical structure and crystallinity of CaP materials could also be tuned to control osteoclast activities. For instance, Davison et al. (2014) indicated that β-TCP with submicron structure feature facilitated human peripheral blood monocytes differentiation into osteoclasts compared to microstructure. Similarly, another study pointed out that the surface structure of BCP affected osteoclastogenesis and bone ectopic formation after material implantation into the dorsal muscle of dogs (Davison et al., 2015). A recent study also showed that osteoclasts could sense the crystallinity of materials and higher crystallinity enhances metabolic and resorption activities of osteoclasts (Uskokovic et al., 2019).

In addition, the phases of CaPs in implanted materials could affect the osteoblasts-osteoclasts crosstalk *in vitro*. Shiwaku et al. (2015) found that osteoclasts cultured on BCPs with low HA content (5%) increased the expression of positive coupling factors, including sphingosine-kinase 1 (SPHK1) and collagen triple helix repeat containing 1 (Cthrc1). In a subsequent study, they cultured osteoclasts on HA/OCP or HA/TCP disks to investigate how the distinct chemical composition and crystal structure affect osteoclast formation and the osteoclasts-osteoblasts crosstalk. Both OCP and β-TCP had a similar ability to create multinucleated osteoclasts. OCP promoted the expression of complement component 3a (C3a) and β-TCP enhanced that of EphrinB2 (EfnB2) and Cthrc1 in osteoclasts, respectively. In turn, these secreted positive coupling factors promoted osteoblast differentiation and function (Shiwaku et al., 2019). Chemical properties, such as dissolution and precipitation around OCP and β-TCP crystals, may be factors that contribute to similar and different cellular responses in these CaP phases.

#### 3.2.3 Biological Cell Responses to Internalized Calcium Phosphate Particles

Nanoscale CaP particles are produced from degradation or mechanical abrasion of macroscopic implants or direct implantation as nanoscale bone substitute materials (Epple, 2018). In all these cases, the surrounding tissue is subjected to calcium phosphate nanoparticles (CaP-NPs) at varying concentrations. Apart from small CaP-NPs (a few nm in diameter) that can directly penetrate the cell membrane on their own, almost all CaP nanoparticles are internalized by endocytosis and/or phagocytosis (Mahmoudi et al., 2011). Primary endosomes or phagosomes then fuse with cytoplasmic lysosomes to dissolve the CaP-NPs into calcium and phosphate. The cargo is eventually delivered into the cytoplasm (Epple, 2018). The CaP-NP-induced apoptotic pathway is mediated *via* a mitochondrial-dependent pathway (Xu et al., 2012; Xue et al., 2017). The sizes (Shi et al., 2009), surface topography and morphology (Zhao et al., 2007; Okada et al., 2010) and concentrations (Xu et al., 2012) of CaP-NPs have significant effects on the apoptotic level. The CaP-NPs are not cytotoxic *per se*, but the release of a large number of calcium ions after dissolution of the CaP-NPs is harmful. And apoptosis is induced by increased intracellular calcium ions concentration (Masouleh et al., 2017).

It is worthy that particles released from CaP materials degradation should not disturb the ability of bone-forming cells to form new bone. Therefore, in addition to studying the effect of materials’ macroscopic properties on the osteogenic ability of MSCs/osteoblasts, more studies on the effects of CaP-NPs on these cells and the underlying mechanisms have been investigated (Albulescu et al., 2019; Khotib et al., 2021). Particle size, shape, and concentration will have different effects on the function, viability, proliferation, and extracellular matrix of osteoblasts (Sheikh et al., 2015; Li et al., 2022). CaP-NPs have dose-dependent effects on osteoblasts and the maximum number of nanoparticles a single osteoblast can tolerate is 50 (Pioletti et al., 2000). Small CaP particles (1–10 μm) are less biocompatible than large particles (>10 μm) on the viability, proliferation and gene expression of osteoblasts (Pioletti et al., 2000). The CaP-NPs with higher specific surface area could increase the adhesion of osteoblasts. However, Xu et al. (2012) found that larger specific surface areas of CaP-NPs might induce higher apoptosis rates in the concentration range of 20–100 μg/ml. Jin et al. (2017) have studied the effect of unfunctionalized calcium phosphate nanorods (HA; 20 nm in diameter and 80 nm in length) on MC3T3 cells. They observed an uptake by micropinocytosis and a significant cytotoxicity above 40 mg L^−1^, which was caused by oxidative stress and lysosomal rupture. In a study using SAOS-2 osteoblasts, nano-HA were actively internalized and are retained within intracellular membrane-bound compartments. Dissolution of the nano-HA was observed within phagolysosomes. After 24 h of internalization, re-precipitation of needle-like minerals appears in the intracellular membrane-bound compartments (Rossi et al., 2018). These re-precipitated nanoparticles may provide a cellular protective mechanism against the cytotoxicity of rapidly elevated cytoplasmic calcium ions (Rossi et al., 2018).

In addition, particle size also significantly affects the activity of stimulating osteogenic differentiation. Yang et al. (2018) cultured MSCs with three different-sized nano-HA (∼50, ∼100, and ∼150 nm, resp.) and found that smaller-sized nano-HA (∼50 and ∼100 nm) accelerated the expression of osteoblast-like cell osteogenic genes (Yang et al., 2018). This is in line with that smaller-sized nano-HA (40–1000 nm) appeared to be more effective in stimulating osteogenic differentiation of hMSCs (Weissenboeck et al., 2006). An *in vitro* enzymatic reaction route has been employed for generating biomimetic amorphous calcium phosphate (EACP) nanominerals (Jiang et al., 2018). The amorphous nanomineral is composed of organic-inorganic hybrid nanoparticles. Adenosine triphosphate (ATP) provides the pool of phosphate ions for EACP formation. After internalization of EACP into human bone marrow-derived mesenchymal stem cells (hBMSCs), the release of ADP/AMP biomolecules and calcium ions activate adenosine 5′-monophosphate (AMP)-activated protein kinase (AMPK) and induces autophagy and osteogenic differentiation of hBMSCs (Jiang et al., 2018).

## 4 Customizable Calcium Phosphate-Polymer Composite Biomaterials

Although humans share a set of bone healing mechanisms, the effects of bone healing vary in individuals, depending on the conditions of bone defects. Such diversities account for the different rates and effects of bone regeneration. Because these biomaterials are likely to be transplanted into an ‘unfriendly’ host, the disease state, and other specifics of the host, such as age, ongoing chronic inflammation and infection should be well understood and taken into consideration. Accordingly, the fabrication of a customizable bone graft to match the condition of the bone defect is important for efficient bone regeneration.

Customizable bone graft substitutes may be designed by following two principles: 1) Tailored external construction and internal structural mimicking of the microenvironment of the defect site. The specific technologies and methods relevant to the design and manufacture of such materials are summarized in another review (Forrestal et al., 2017). 2) Customized biodegradation rate that matches the new bone formation rate, which needs to be complemented by an appropriate *in vivo* bone healing response. In this context, introducing CaPs into smart hydrogel systems may be a promising solution.

Hydrogels possess good biocompatibility and tissue integration properties and serve as the carriers in drug systems and bone tissue engineering biomaterials (Forrestal et al., 2017; Mantha et al., 2019). In response to stimulus [including exogenous (thermoresponsively-, magnetically-, electroresponsively- or light-triggered) and internal physical/biochemical (pH-, redox- or enzyme-sensitive)], hydrogel systems can change their structures, compositions or conformations to support the release of encapsulated active species (Claassen et al., 2019; Wei et al., 2022). The specific microenvironment of bone healing process, such as specific immune environment or enzyme release, can be used to design corresponding intelligent stimulus-responsive biomaterials for rapid reactive bone therapy and bone regeneration. Enzyme-responsive systems provide enlightenments for the selection of biomolecules. The design of enzyme-responsive systems for drug delivery relies on esterase- or protease-mediated cleavage of esters or short peptide sequences, resulting in effective drug release (Ramasamy et al., 2017). By choosing an appropriate peptide sequence, enzyme-responsive materials may be designed to respond exclusively to a target enzyme (Ramasamy et al., 2017). To be specific, tissue-nonspecific alkaline phosphatase (TNAP) may be considered a candidate. The TNAP hydrolyzes inorganic pyrophosphate (PPi, a potent mineralization inhibitor) and ATP into monophosphate (Pi, inorganic phosphate) to control ECM mineralization (Bottini et al., 2018). Ideally, a matching peptide sequence can make polymers respond exclusively to TNAP. New bone formation that coordinates with the biodegradation of CaP-polymer composites may be achieved by harnessing the TNAP-response to achieve a customized biodegradation rate.

## 5 Conclusion

**FIGURE 2:**
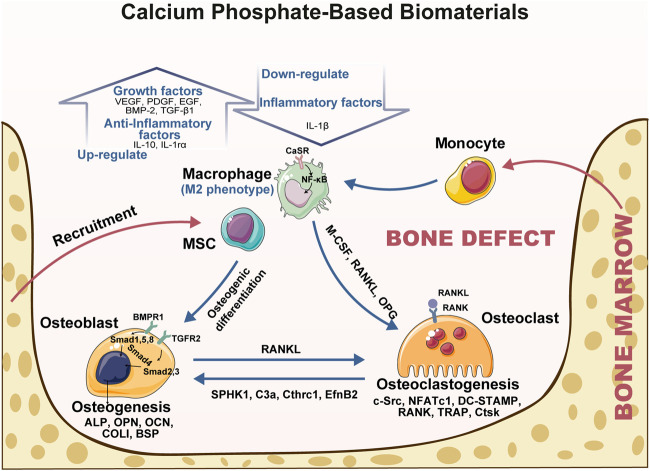
The interactions between CaP-based biomaterials and bone healing response. The osteoinductivity of CaP-based biomaterials can be “programmed” by their physico-chemical properties and are necessary for modifying the behavior of immune cells, osteoclasts, and MSCs/osteoblasts. Under the simulation with CaP-based biomaterials, the expression of inflammatory factors (IL-1β) is decreased, and the production of anti-inflammatory cytokine (IL-10, IL-1rα), as well as growth factors (VEGF, PDGF, EGF, BMP-2, and TGF-β1) is increased in macrophages. The surface structure and crystallinity of CaP-based biomaterials can be sensed by osteoclasts and regulate their metabolically active and resorption capacity. All above synergistically create a pro-osteogenic micro-environment leading to effects on bone formation. Abbreviation: ALP, alkaline phosphatase; BMP-2, bone morphogenetic protein two; BSP, bone sialoprotein; CaSR, calcium-sensing receptor; C3a, complement component 3a; Cthrc1, collagen triple helix repeat containing one; COL-I, collagen I; Ctsk, cathepsin k. DC-STAMP, dendritic cell-specific transmembrane protein; EfnB2, EphrinB2; EGF, epidermal growth factor; IL, interleukin; SPHK1, sphingosine-kinase one; MCP-1, monocyte chemoattractant protein-1; M-CSF, macrophage colony-stimulating factor; NFATc1, nuclear factor of activated T cells one; OCN, osteocalcin; OPN, osteopontin; OPG, osteoprotegerin; PDGF, platelet derived growth factor; RANKL, receptor activator of NF-κB ligand; TGF-β, transforming growth factor beta; TNF-α, tumor necrosis factor-α; TRAP, tartrate-resistant acid phosphatase; VEGF, vascular endothelial growth factor.

Synthetic biodegradable bone grafts are gradually being replaced by the patient’s own bone. After implantation, tight and complex interactions occur between CaP-based biomaterials and the bone healing process. Multiple cells involved in bone healing mediate the materials’ degradation and resorption, which is essential to provide the space into which the bone and vascular tissues can grow. The physico-chemical properties of materials are also necessary for modifying the behavior of cells/tissues and enhancing the osteoinductivity of the biomaterials. In light with previous studies, we summarized the osteoinductive mechanism of CaP-based biomaterials accompanying bone healing (Figure 2). Under the stimulation with CaP-based biomaterials, the expression of inflammatory factors (IL-1β) is decreased, and the production of anti-inflammatory cytokine (IL-10, IL-1rα), as well as growth factors (VEGF, PDGF, EGF, BMP-2 and TGF-β1) is increased in macrophages, which synergistically create a pro-osteogenic micro-environment. The surface topography and crystallinity of CaP-based biomaterials can be sensed by osteoclasts and regulate their metabolic activity and resorption capacity, then influencing bone formation. These material particles are eventually internalized into cells and are involved in the regulation of cell homeostasis and cell differentiation. Based on the in-depth understanding of the involvement of CaP-based biomaterials in the bone healing process, we propose the future design of these materials is to develop biomaterials with customizability, such as tailored geometry and biodegradation rate.
